# Intestinal-Targeted Digestion of Heme Chloride by Forming Inclusion Complexes In Vitro

**DOI:** 10.3390/foods13193078

**Published:** 2024-09-27

**Authors:** Qianfan Yu, Li Huang, Yuemei Zhang, Wendi Teng, Ying Wang, Jinxuan Cao, Jinpeng Wang

**Affiliations:** 1 Key Laboratory of Geriatric Nutrition and Health, Beijing Technology and Business University, Ministry of Education, Beijing 100048, China; yuqianfan02@163.com (Q.Y.); 2130022073@st.btbu.edu.cn (L.H.); zhangyuemei@btbu.edu.cn (Y.Z.); wenditeng@btbu.edu.cn (W.T.); wang-ying@btbu.edu.cn (Y.W.); caojinxuan@btbu.edu.cn (J.C.); 2 School of Food and Health, Beijing Technology and Business University, Beijing 100048, China; 3 College of Food and Biological Engineering, Chengdu University, Chengdu 610106, China

**Keywords:** hemin, iron fortifier, cyclodextrin, solubility

## Abstract

Hemin, a heme-like compound with significant biological activity, shows promise as an iron supplement for humans. Nonetheless, its poor solubility in water greatly impedes its absorption and utilization. To surmount this obstacle, researchers have chosen various cyclodextrins with distinct cavity sizes and derivative groups to act as hosts, forming inclusion complexes with hemin chloride. Among these, γ-cyclodextrin has been identified as the optimal carrier, based on a thorough evaluation of its encapsulation efficiency, solubility, and molecular docking. Multiple characterization techniques further confirmed the formation of these inclusion complexes. Results from IEC-6 cell experiments indicated that the cytotoxicity of the inclusion complexes was lower than that of FeSO_4_. Static and dynamic gastrointestinal simulation digestion systems were established, and the results showed that the bioavailability of the inclusion complex was significantly higher than that of raw hemin. Additionally, only about 0.29% of hemin chloride is digested by gastric enzymes, whereas 9.52% is digested by pancreatic enzymes in the static gastrointestinal simulation digestion system, with similar outcomes observed in the dynamic system. These findings suggest that targeted digestion in the intestine significantly enhances the bioavailability of hemin chloride by forming inclusion complexes in vitro.

## 1. Introduction

Heme iron is mainly extracted from hemoglobin by enzymatic hydrolysis, but it often exists in the form of hemin [1,2]. Iron (III) protoporphyrin IX chloride, as a stable form of heme iron, has been listed as a nutritional fortifier [3], which can be used to supplement iron for human body. It is reported that hemin can obviously improve anemia in the treatment of iron deficiency anemia in children. Hemin not only has remarkable curative effect and high cure rate but also has no gastrointestinal side effects [4,5]. However, the absorption and utilization of hemin is greatly limited, mainly because of its poor dispersion in water. At present, the commonly used solutions for the improvement of its water dispersibility include direct encapsulation and chemical derivation. Among them, the chemical derivative method has some problems, such as complex operations, many by-products, hidden dangers of food safety, and so on. The physical encapsulation method is safe, easy to prepare, and environment-friendly. Thus, researchers prefer physical encapsulation to improve its water dispersibility. Nanoparticles have emerged as promising candidates for encapsulation purposes. Their application in encapsulating proteins, polysaccharides, and their complexes has been found to enhance the water dispersibility of hemin [6,7,8,9]. A noteworthy example is the soybean isolate protein complex nanoparticle, which demonstrates superior solubility compared to free hemin [10]. Polymeric micelles were used to encapsulate hemin [11], and water-soluble sodium alginate (SA) was used to produce heme iron-alginate beads [12,13]. Alternatively, heme iron could be delivered via liposome loading for iron fortification purposes [14,15]. Furthermore, microencapsulation of heme or hemin using maltodextrin encapsulation has been explored by certain researchers to enhance their absorption rates [16,17].

Cyclodextrins (CDs) are a group of cyclic oligosaccharides formed by glucose units linked together with α-1,4 glycosidic bonds [18,19]. Due to its hydrophilic exterior and hydrophobic interior cavity, it facilitates the formation of inclusion complexes with diverse hydrophobic guest molecules, thereby enhancing the solubility of the substance in water [20]. The encapsulation of cyclodextrins has garnered increasing attention due to their favorable biocompatibility, minimal toxicity, distinctive molecular structure, and straightforward operation [21]. Despite numerous studies employing cyclodextrins as delivery systems for bioactive compounds, limited research has been conducted on their application as encapsulating agents for oral iron supplementation [22]. Currently, the encapsulation of hemin by β-cyclodextrin is the only reported instance [23]. It is noteworthy that the solubility of β-cyclodextrin stands at a mere 1.8 g per 100 mL of water, resulting in poor dispersion of the inclusion complex within the aqueous medium. Consequently, obtaining the desired iron supplementation effect from the same dosage of the iron agent becomes challenging. To address this, it is imperative to meticulously select an appropriate cyclodextrin that can effectively enhance the dispersion of hemin in water and its bioavailability.

The encapsulation properties of cyclodextrins, which vary in cavity size and derivative groups, are markedly distinct. Molecular docking (MD) serves as a crucial instrument for identifying the most suitable cyclodextrin for subsequent research or application [24]. Farhad et al. have shown that methyl β-Cyclodextrin, with its randomly substituted groups, exhibits the highest binding affinity for Ataluren, while α-Cyclodextrin displays the weakest affinity, as indicated with MD simulations [25]. Ramesh et al. found that the inclusion complexes of antipyrine with β and γ-cyclodextrin are more stable than those with α-cyclodextrin, based on their MD analysis [23]. In a separate study, it was revealed that levopromazine has a particularly high binding affinity for HP-β-CD, surpassing its affinity for other cyclodextrins, as demonstrated with MD simulations [26]. Nevertheless, hemin, a well-established iron supplement, has not been extensively studied.

In the present study, hemin-cyclodextrin inclusion complexes were produced using the solution stirring method. Based on the outcomes of entrapment efficiency, solubility, and molecular docking assessments, the most suitable cyclodextrin was selected. The obtained inclusion complexes were characterized using different analytical techniques, including Fourier transform infrared spectroscopy (FTIR), X-ray diffraction (XRD) analysis, and scanning electron microscope observation (SEM). Furthermore, influence of the cyclodextrin encapsulation on the bioavailability of hemin was also investigated by using an in vitro, simulated gastrointestinal digestion model. This research could provide a delivery carrier for hemin, not only improving the water-solubility and bioavailability of hemin but also supporting the development of some iron-fortified nutraceutical foods.

## 2. Materials and Methods

### 2.1. Materials

Hemin with purity above 98% was obtained from Mreda Co., Ltd. (Beijing, China). β-cyclodextrin (β-CD), methyl-β-cyclodextrin (Me-β-CD), hydroxypropyl-β-cyclodextrin (HP-β-CD), sulfobutyl ether-β-cyclodextrin (SBE-β-CD), and γ-cyclodextrin (γ-CD) with purity above 98% were obtained from Zhiyuan Biotechnology Co., Ltd. (Binzhou, China). γ-CD with purity above 98% was obtained from Shanghai yuanye Biotechnology Co., Ltd. (Shanghai, China). IEC-6 cells were obtained from Shanghai Fuhang Biotechnology Co., Ltd. (Shanghai, China). Other chemical reagents are of analytical grade and purchased from Mreda Co., Ltd. and Modern Oriental Technology Development Co., Ltd. (Beijing, China).

### 2.2. The Preparation of Inclusion Complexes of Hemin with CDs

The inclusion complexes of hemin with CDs (β-CD, Me-β-CD, HP-β-CD, SBE-β-CD, and γ-CD) were prepared using the method described by Yuan Xi [23]. The hemin and CDs were weighted with a 1:2 molar ratio; the hemin was dissolved in a small amount of dilute ammonia water, and the CDs were dissolved in distilled water. The aqueous cyclodextrin solution was stirred using a magnetic stirrer, and the hemin solution was slowly added to the aqueous cyclodextrin solution. The mixed solution was stirred continuously at 40 °C for 3 h for the inclusion reaction. Then it was dried and washed with acid acetone 2 times to remove the free hemin. After the acetone was volatilized, the corresponding inclusion complexes were obtained by drying.

### 2.3. The Preparation of Physical Mixture of Hemin with CDs

Hemin and cyclodextrin were weighed separately at a molar ratio of 1:2; the vortex oscillated for 1 min and was mixed thoroughly to obtain a physical mixture of hemin and cyclodextrin.

### 2.4. Determination of Encapsulation Efficiency (EE)

The EE of hemin was determined according to the method described in the reference [23]. The inclusion complexes were dissolved in a 0.1 mol/L sodium hydroxide solution and was diluted into a 100 mL flask and was shaken well. An accurate amount of 1 mL was taken and placed in a 50 mL measuring bottle, which was diluted with a 0.1 mol/L sodium hydroxide solution to scale and was shaken well. Meanwhile, a standard curve of hemin standards was made in a concentration range of 2–10 ug/mL in 0.1 mol/L sodium hydroxide solution. The inclusion complexes and the standards were measured using UV spectrophotometry at a wavelength of 385 nm, and the concentration of encapsulated hemin was determined using the standard curve.

The encapsulation efficiency (EE) was calculated according to Equation (1):

(1) $$\mathrm{EE}\;(\%)=\;\frac{{Hmn}_{exp}(\mathrm{mg})}{{Hmn}_{T}(\mathrm{mg})}\times 100$$

where *Hmn_exp_* presents the encapsulated amount of hemin, and *Hmn_T_* presents the initial amount of hemin.

### 2.5. Determination of Solubility

The excess inclusion complexes were dissolved with 10 mL of distilled water and was vortex oscillated to mix well. Then, it was kept away from light for 24 h at room temperature to equilibrium. The solution was centrifuged at 5000 rpm for 10 min, and the precipitation was removed by filtration. Then, the filtrate of 1 mL was diluted properly with 0.1 mol/L of sodium hydroxide. UV spectrophotometry determined the solubility of the inclusion complexes. The solubilization ratio was calculated with hemin as the control sample.

The solubility and solubilization ratio were calculated by using the following equations:

(2) $$\mathrm{Solubility}\;(\mu g/\mathrm{mL})=\;\frac{m_{Hmn}(\mathrm{ug})}{V(\mathrm{mL})}\times 100$$

(3) $$\mathrm{Solubilization}\;\mathrm{ratio}=\frac{S_{exp}(\mu g)}{S_{Hmn}(\mu g)}\times 100$$

where *m_Hmn_* presents the amount of hemin dissolved, and *V* presents the total volume of the solution in Equation (2). *S_exp_* presents the solubility of encapsulated hemin, and *S_Hmn_* presents the solubility of original hemin in Equation (3).

### 2.6. Molecular Docking

Molecular docking simulations were conducted employing the Autodock Tools (Vertion 1.5.7) software to explore the configurations of the inclusion complexes. The crystal structures of β-CD and γ-CD were procured and extracted from the Cambridge Crystal Database. Subsequently, the 3D structures of the corresponding derivatives on the β-CD molecule, namely Me-β-CD, HP-β-CD, and SBE-β-CD, were generated using ChemBio3D Ultra 15.0 and subsequently optimized for energy minimization through an MM2 force field protocol. The 3D structure of hemin was sourced from the PubChem database. Autodock Tools 1.5.7 was utilized to predict the binding modes and binding energies between the host cyclodextrins (CDs) and the guest molecule hemin. During the simulation, hemin was designated as flexible, while the CDs were treated as rigid. A grid box of dimensions 40 × 40 × 40 points with a spacing of 0.375 Å was established for the CDs. The Lamarckian Genetic Algorithm (LGA) was implemented to simulate the molecular docking process, and the conformation exhibiting the lowest binding energy was selected as the optimal complex.

### 2.7. Characterization of the Inclusion Complexes

#### 2.7.1. Fourier Transform-Infrared Spectroscopy (FTIR)

FTIR measurements were conducted with a Nicolet IS10 Intelligent Fourier infrared spectrometer ((Nicolet is50, Thermo Electron Corporation, Waltham, MA, USA). The hemin, γ-CD, the physical mixtures, and the inclusion complexes were ground and mixed with KBr. Then, the samples were pressed into flakes and placed in the light path to obtain the FT-IR spectrum. All analyses were recorded from 400 to 4000 cm^−1^.

#### 2.7.2. X-ray Diffractometry (XRD)

XRD measurements were determined by using the X-ray diffractometer (A24A10, BRUKER AXS GmbH Co., Karlsruhe, Germany) with the following determination conditions: Co-Ka (k = 1.79026 A) radiation, operating voltage 40 KV, output current 40 mA, diffraction angle scanning range of 5°~60°, and scanning speed of 5°/min. 

#### 2.7.3. Scanning Electron Microscopy (SEM)

The morphology of the tested samples was recorded using a scanning electron microscope system (SU8010, HITACHI, Japan). The samples were attached to the SEM aluminum stubs via 2-sided adhesive tape. The powders were treated with gold spraying at an accelerated voltage of 15.0 kV. The appearance of the sample was scanned to observe the morphology of the sample before and after inclusion.

### 2.8. Cultures of IEC-6 Cells

According to the research of Thomas [27], intestinal cell line 6 (IEC-6) cells were cultured and counted in four big compartments. The calculating formula read as follows:

Cell number (cells/mL) = total number of cells/4 × 10^4^ (4)

### 2.9. Cytotoxicity Assay of IEC-6 Cells

The cytotoxicity test was carried out using the cell counting kit-8 (CCK-8) method. The CCK-8 method is a highly sensitive, non-radioactive, colorimetric assay for the determination of the number of viable cells in cell proliferation and cytotoxicity assays. The IEC-6 cytotoxicity assay involved diluting two groups of master mixes to iron concentrations of 0.5 µg/mL, 1.0 µg/mL, 1.5 µg/mL, 2.0 µg/mL, and 2.5 µg/mL using a complete medium. IEC-6 cells were injected in 96-well plates at 5 × 10^4^ cells/mL, as described before. The blank group contained only a culture medium, whereas the control group contained a culture medium but no sample. The optical density of the sample was measured at 450 nm.

Cell viability inhibition rate = (OD_sample_ − OD_blank_/OD_control_ − OD_blank_) × 100% (5)

### 2.10. Simulated Gastrointestinal Digestion In Vitro

The prepared inclusion complexes were subjected to an in vitro digestion model, which will mimic the digestion in the stomach and intestines for the release of hemin from the inclusion complexes.

#### 2.10.1. Static Digestion Simulation

A total of 10 mg of the inclusion complex was dissolved in 5 mL of distilled water with ultrasound for 5 min. For gastric digestion, 10 mL of simulated gastric juice (SGJ) was added into 5 mL of aqueous solution and vortex oscillated to mix well. The mixture consisting of the sample and the SGJ was subjected to incubation at 37 °C at a speed of 160 r/min in a temperature-controlled automatic shaker. Intestinal digestion was performed after 2 h of incubation, and pH = 7 was adjusted with 2 M NaOH. Then, 10 mL of simulated intestinal juice (SIJ) was added into the above mixed solution and vortex oscillated to ensure it was evenly mixed. Then, the mixture was again incubated at 37 °C at a speed of 160 r/min in a temperature-controlled automatic shaker for 4 h.

At the end of the reaction, part of the gastric digestive products (GD) and part of the gastrointestinal digestive products (GID) were centrifuged at 10,000 g for 10 min, and the supernatants were collected for the determination of hemin retained in the aqueous phase after digestion in vitro, while the other parts of GD and GID were directly used to determine the content of hemin in the whole digestive juice.

#### 2.10.2. Dynamic Digestion Simulation

Dynamic digestion simulation was performed in the BGR bionic gastrointestinal reactor, a multicompartment dynamic digestive system simulator, which consists of three connected reactors representing the different parts of the human gastrointestinal tract. The experiment was performed using only the first two reactors, a bionic stomach reactor and a small intestine reactor. Prior to the in vitro digestion, 45.33 mg of the inclusion complex was dissolved in 50 mL of water to prepare the inclusion complex solution.

The BGR bionic gastric reactor module can hold up to 200 mL of digestive juice, so the 50 mL inclusion complex solution was mixed with 150 mL of artificial gastric juice and injected with 200 mL. At first, 10% of the volume of the simulated gastric juice (SGJ) was pumped into the BGR reactor, and then the inclusion complex solution was pumped into the reactor. The inflow rate of the SGI started at 0.6 mL/min and slowly increased to 3.2 mL/min within 40 min and then 40–60 min down to 0.6 mL/min, ensuring that the ratio of the final sample for the artificial gastric juice was 1:3. In this process, the frequency of gastric peristalsis was 3 times/min. The BGR system was maintained at 37 °C, and the pH was automatically adjusted to around 2.0. After 2 h of digestion, 5 mL samples were taken out for follow-up measurement.

After gastric digestion, the pH value was adjusted to about 6.0 with 1.0 M NaHCO_3_. Then the gastric contents were gradually transported to the bionic intestine reactor through the peristaltic pump at the end of the gastric reactor. Next, 50 mL intestinal juice was pumped into the reactor and mixed with the product of gastric digestion. The pH value was automatically adjusted by pumping the NaOH solution (0.5 mol/L). Next, 5 mL samples were taken after being digested for 0.5 h at a pH of 6.0. Then, the pH was adjusted to 6.5 and digested for 1.5 h. Finally, the pH was adjusted to 7. 0, digested for 2 h, and sampled for subsequent measurements. The gastrointestinal simulation digestion in vitro of Hmn and the mixture was consistent with the above steps; only the inclusion complex solution was replaced with the same amount of Hmm and the mixture solution, respectively. The collected samples were centrifuged at 5000 r/min for 10 min to obtain the supernatant and diluted with 0.1 mol/L of NaOH. The content of Hmn was calculated by measuring the absorbance value at a 385 nm wavelength.

### 2.11. Statistical Analysis

All experiments were repeated three times, and the results were presented as means ± standard deviation (SD). The data were analyzed using one-way analysis of variance (ANOVA) and Duncan’s multiple range test in SPSS 14.0 software. Additionally, data visualization graphs were created using Origin 2021 software.

## 3. Results and Discussion

### 3.1. Cyclodextrin Selection by MD

**Figure 1:**
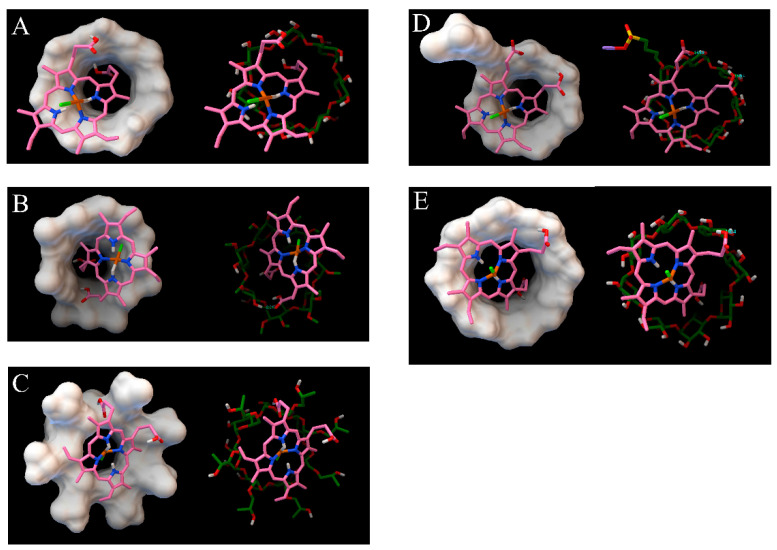
The molecular docking of hemin/β-CD IC (**A**), hemin/Me-β-CD IC (**B**), hemin/HP-β-CD IC (**C**), hemin/SBE-β-CD IC (**D**), and hemin/γ-CD IC (**E**) (short green line indicates intermolecular hydrogen bond).

The intermolecular interactions between cyclodextrin and its guest molecules, which encompass hydrogen bonds, van der Waals forces, and hydrophobic interactions, are crucial for stabilizing the structures of inclusion complexes. These interactions, however, vary significantly depending on the size and type of substituent groups present in the cyclodextrin molecules [28]. The results depicted in Figure 1 indicate that the hemin side chain has entered the hydrophobic cavity of the cyclodextrins (CDs) via the wider opening, adopting a semi-inclusion configuration with the CDs. The remainder of the hemin side chain lies along the periphery of the CDs’ cavity. Additionally, hydrogen bonds between the host and guest molecules contribute to the stabilization of the supramolecular structure. The binding energies between hemin and CDs ranged from −4.22 kCal/mol to −5.18 kCal/mol. It is noteworthy that a lower binding energy in MD corresponds to a more stable inclusion complex formation. The ability of various cyclodextrins (CDs) to include hemin follows the order γ-CD > HP-β-CD > SBE-β-CD > Me-β-CD > β-CD. Factors influencing the formation of cyclodextrin inclusion complexes encompass geometric size, the polarity of the guest molecules, and the extent of spatial steric hindrance [29,30,31]. However, the findings from molecular dynamics simulations in this study indicate that geometric size compatibility is the most critical factor.

### 3.2. Encapsulation Efficiency Analysis

Encapsulation efficiency (EE) was assessed via ultraviolet spectroscopy to indicate the level of drug entrapment by the carrier, with the corresponding data presented in Table 1. The hemin EE of the hemin/β-CD IC, hemin/Me-β-CD IC, hemin/HP-β-CD IC, hemin/SBE-β-CD IC, and hemin/γ-CD IC were 70.91 ± 0.86%, 70.24 ± 0.61%, 71.67 ± 1.23%, 68.17 ± 0.58%, and 72.11 ± 0.08%, respectively. The enhanced encapsulation can be attributed to the abundant availability of CDs, which facilitates the inclusion process. Soγ-CD was verified as the most suitable cyclodextrin due to its highest encapsulation efficiency (EE). This finding highlights the reliability of molecular docking technology in screening cyclodextrins for optimal encapsulation efficiency.

### 3.3. Dispersibility Analysis

The dispersion properties are intrinsically linked to the bioavailability and uptake efficiency of functional components. The water dispersibility of the five inclusion complexes, as outlined in Table 2, spans from 21.01 ± 1.82 to 163.61 ± 1.78 ug/mL. In contrast, the water dispersibility of hemin is significantly lower, at 0.68 ug/mL, indicating its near insolubility. The encapsulation of β-CD, Me-β-CD, HP-β-CD, SBE-β-CD, and γ-CD has resulted in a marked improvement in hemin solubility, with increases ranging from 30.90 to 240.60 times. The successful embedding of guest molecules for solubilization purposes necessitates a careful consideration of both the compatibility between the guest and host molecules, as well as the solubility of the embedding host molecule itself [32].

### 3.4. Characterization of the Inclusion Complexes

#### 3.4.1. FTIR

**Figure 2:**
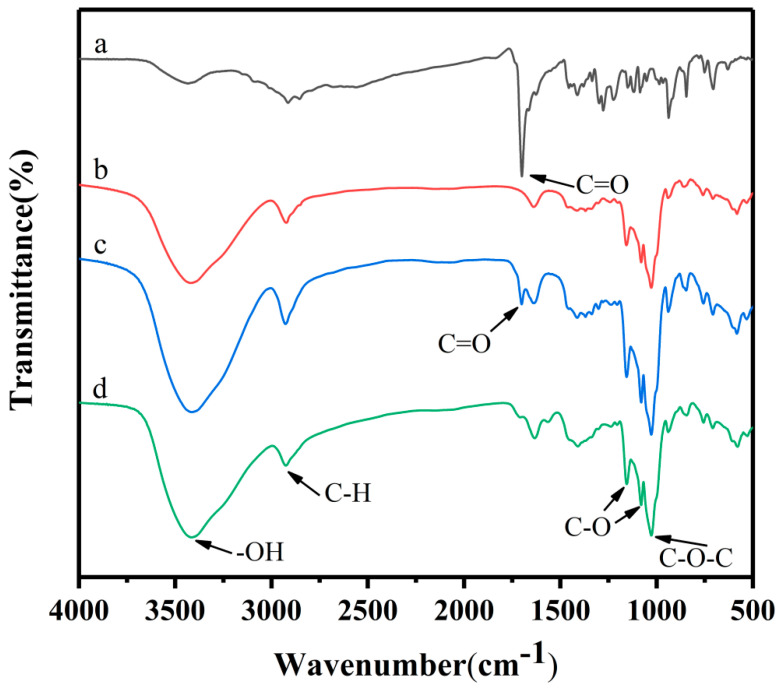
FTIR spectrum of (a) hemin, (b) γ−CD, (c) physical mixture, and (d) inclusion complex.

The FTIR spectra of hemin, γ-CD, their physical mixture, and the inclusion complex are depicted in Figure 2. The spectra reveal a characteristic absorption peak at 3435.16 cm^−1^, indicative of the -O-H bond stretching, and another at 2914.83 cm^−1^, which corresponds to the -C-H bond stretching. The peak at 1700.08 cm^−1^ signifies the C=O stretching vibration on the carbonyl group [10]. γ-CD exhibits a broad characteristic band for the -O-H stretching at 3419.06 cm^−1^ and a C-O-C stretching vibration peak at 1028.14 cm^−1^. The infrared spectrum of the physical mixture displays a mere superposition of the absorption peaks from both hemin and γ-CD, suggesting that hemin has not been encapsulated within γ-CD [33]. In contrast, the spectrum of the hemin/γ-CD inclusion complex shows the disappearance of the C=O stretching vibration peak at 1700.08 cm^−1^, aligning closely with the pattern of γ-CD alone [10]. This suggests that the small molecules of hemin have entered the cavities of γ-CD, resulting in the characteristic peaks of hemin being obscured by those of γ-CD. Consequently, the formation of the inclusion complex can be inferred.

#### 3.4.2. XRD

**Figure 3:**
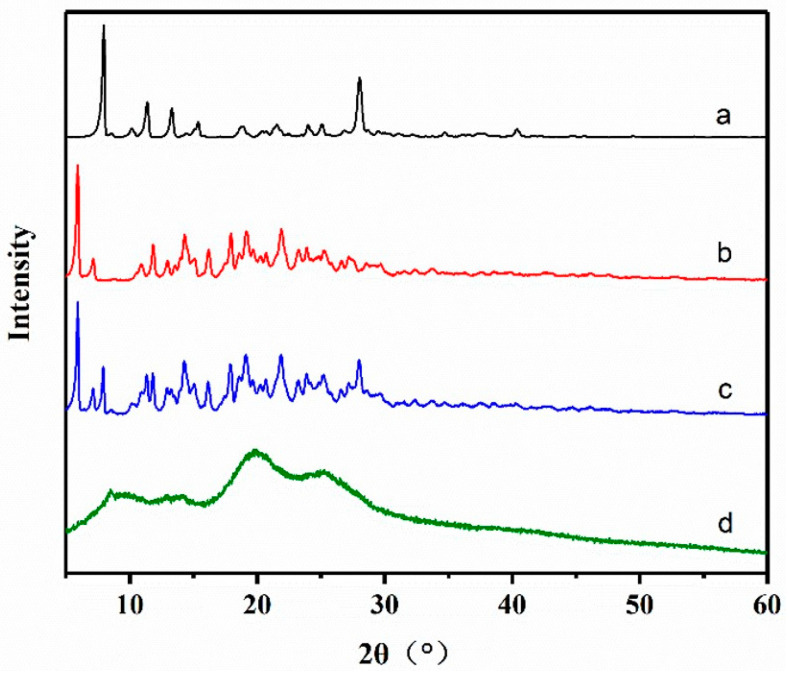
X-ray diffractometry spectra of (a) hemin, (b) γ-CD, (c) physical mixture, and (d) inclusion complex.

The XRD patterns of hemin, γ−CD, the physical mixture, and the inclusion complex are depicted in Figure 3. The XRD patterns of both hemin and γ−CD exhibit a series of distinct sharp diffraction peaks, suggesting that they possess crystalline structures [23]. The obvious characteristic peaks of hemin at 2θ° were 7.96°, 11.39° and 28.03°, while γ−CD had several continuous diffraction peaks at 2θ = 10°~30°, and its characteristic peaks were 5.92°, 7.14°, 11.84°, 14.32°, and 16.20°. The diffraction curve 4c of the physical mixture displayed a diffraction profile similar to that of γ-CD, coupled with distinct crystallization peaks indicative of hemin. This suggests that the diffraction patterns of γ-CD and hemin were merely superimposed, without any evidence of complex interactions forming [34].

The XRD diffraction pattern of the inclusion compound exhibited a complete absence of characteristic peaks associated with hemin. Additionally, there was a significant reduction in diffraction peaks and a notable decrease in the degree of crystallization. Notably, the presence of broadened diffraction peaks indicated a transition from a crystalline state to an amorphous state for hemin [35]. These observations suggest that the structure of the prepared inclusion complex differs significantly from a simple physical mixture. It is hypothesized that hemin may be dispersed within the cyclodextrin cavity, leading to a reduction in crystallinity and the formation of a fully amorphous structure. Amorphous solids have higher molecular mobility and energy than crystal forms. This makes the amorphous system have a higher apparent solubility and dissolution rate, which indirectly verifies the high water solubility of the inclusion complex [32]. 

#### 3.4.3. SEM

**Figure 4:**
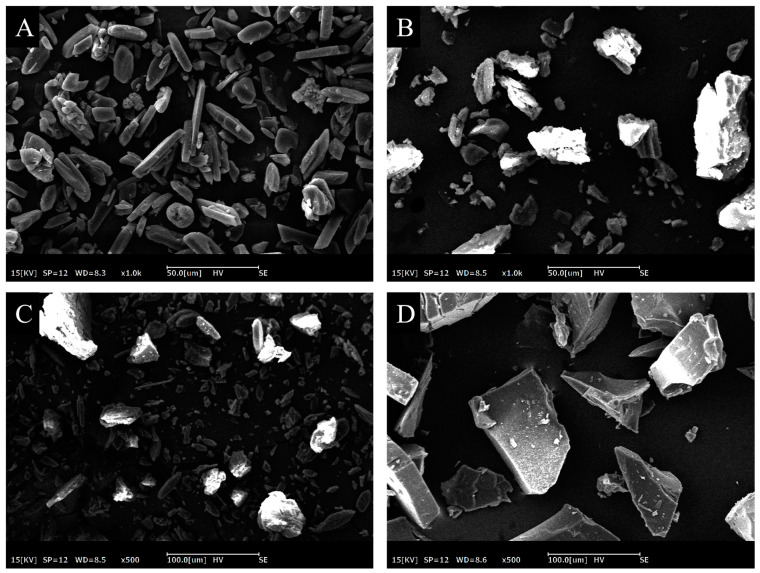
SEM of hemin (**A**), γ-CD (**B**), hemin/γ-CD mixture (**C**), and hemin/γ-CD complex (**D**).

Scanning electron microscopy was an effective tool to observe the external morphology of the solid samples. Figure 4 shows the surface morphology of hemin, γ-CD, Hmn/γ-CD mixture, and the complex. Hemin presented as a uniformly shaped crystal with a sleek exterior, whereas γ-CD displayed a loosely packed, prismatic structure varying in size. When combined physically, the mixtures of hemin and γ-CD demonstrated a basic admixture while maintaining the inherent form of hemin. As shown in Figure 4D, the Hmn/γ-CD IC presented a uniform and irregular, flaky structure. Meanwhile, the original morphology of Hmn and γ-CD disappeared. The transformation in the particle morphology demonstrated a significant distinction in the arrangement of the inclusion complex versus the physical mixture, thereby confirming the formation of hemin-γ-CD inclusion complexes.

### 3.5. Cytotoxicity Assay of IEC-6 Cells

**Figure 5:**
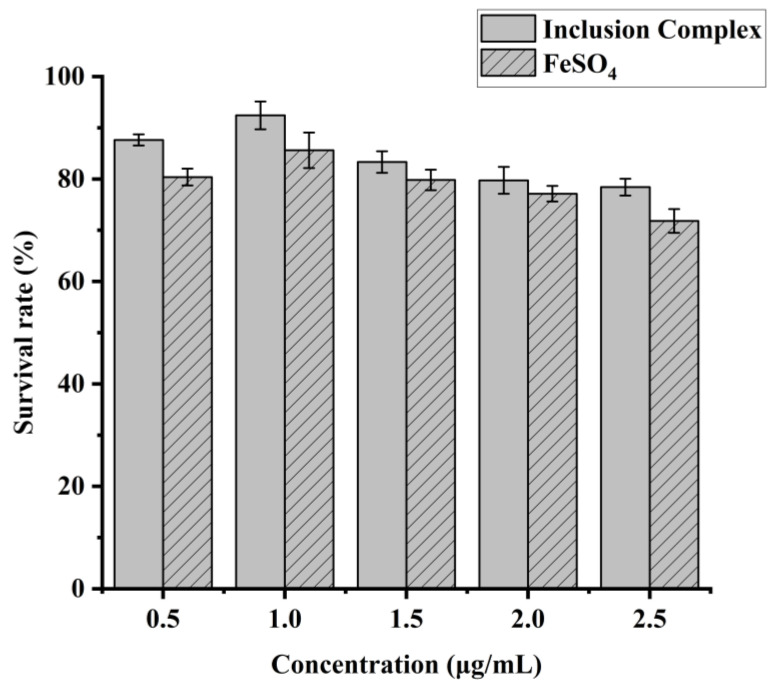
Cytotoxicity of different iron concentrations in IEC-6 cells.

In the context of ensuring the safety of newly developed heme chloride/γ-CD inclusion systems, it is crucial to ascertain their potential toxicity. Therefore, a rigorous evaluation of the cell viability of inclusion complexes have been conducted using IEC-6 cells (Figure 5). Results showed that the best survival rate of IEC-6 cells, around 90% at the dose of 1.0 µg/mL, and IEC-6 cells reduced as iron content increased. This result is consistent with Wang’s findings [36]. Interestingly, at the same dosage, the inclusion complex showed significant less toxicity to Caco-2 and IEC-6 cells than FeSO_4_.

### 3.6. In Vitro Digestion of Hemin

**Figure 6:**
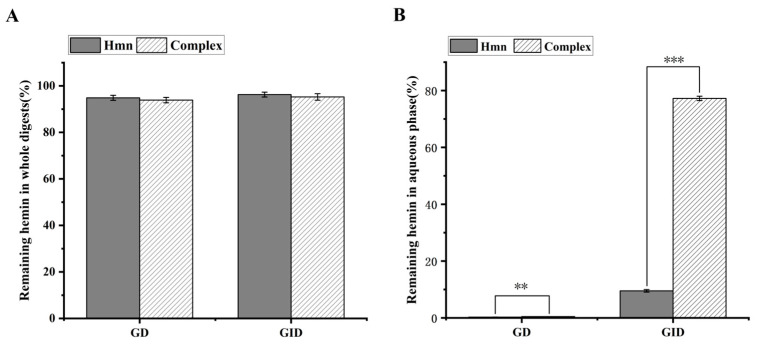
Percentage of hemin remaining in the whole digestion (**A**) and transferred to the aqueous phase (**B**) for free hemin and hemin/γ-CD inclusion complex after simulated gastric digestion and gastric-intestinal digestion (**: *p* < 0.01; ***: *p* < 0.001).

The assessment of the digestive stability and bioavailability of hemin encapsulated within cyclodextrin serves as a critical metric in evaluating its biological functionality. Therefore, the current study employed a simulated gastrointestinal digestion protocol to quantify the stability and bioavailability of hemin throughout the digestion process, encompassing both gastric digestion (GD) and gastrointestinal digestion (GID). The results of this experiment are graphically represented in Figure 6.

From Figure 6A, it can be observed that more than 90% of hemin was not degraded after simulated digestion without a significant difference between the two samples. This finding indicates that hemin exhibits remarkable stability throughout the gastrointestinal digestion process. Alternatively, the bioavailability of hemin is assessed through the quantification of its retention in the aqueous phase, as shown in Figure 6B. Only 0.29% hemin was dissolved in the aqueous phase after gastric digestion (GD) of free hemin; the hemin in the aqueous phase reached 9.52% after trypsin digestion (GID), indicating that the bioavailability of free hemin was very low after being ingested by the human body. Although the content of hemin in the aqueous phase was low (approximately 0.46%) due to the action of gastric enzymes, the encapsulation of hemin within γ-CD formed an inclusion complex that significantly enhanced its bioavailability. Specifically, after trypsin digestion, the bioavailability of hemin encapsulated in γ-CD increased to 77.24%, representing an eightfold increase compared to hemin alone. Furthermore, the bioavailability of hemin is influenced by pH levels within the body during the gastrointestinal digestion process. Notably, the bioavailability of hemin in the neutral environment of intestinal digestion was superior to that observed in the acidic environment of gastric digestion. Consequently, the encapsulation of hemin with γ-CD significantly improved its bioavailability, potentially due to the high water solubility of the resulting inclusion complex.

**Figure 7:**
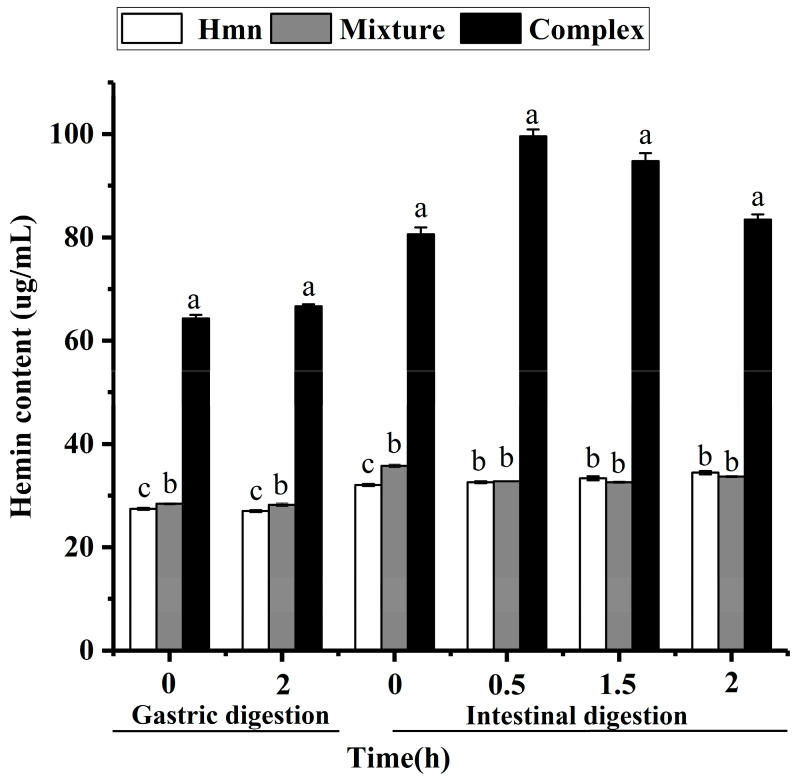
The content of hemin in the digestive juice. Different English letters indicate that there are significant differences between samples.

In the dynamic digestion simulation experiment, the bioavailability was directly reflected by the content of hemin in the digestive juice. The result of this experiment is represented in Figure 7.

A small amount of hemin was released after 2 h of gastric digestion, as shown in Figure 7. This indicates that hemin is stable in gastric juice and can reach the small intestine. After simulated intestinal digestion, the content of hemin in the inclusion complex increased and gradually decreased to 83.49 ug/mL. Compared with the content of free hemin in digestive juice (34.44 ug/mL), the content of hemin in the inclusion complex was significantly increased by 2.4 times. However, there was no significant difference between physical mixture and hemin. Thus, the results showed that, after cyclodextrin encapsulation, the bioavailability of hemin was improved to some extent. At the same time, it was also found that the effect of intestinal digestion was significantly better than that of gastric digestion, which may be related to the pH value of the body. This result is consistent with static simulation digestion.

## 4. Conclusions

To enhance the water solubility and bioavailability of hemin, cyclodextrins (CDs) were selected as prospective carriers, leveraging their distinctive cavity structure and capacity to establish stable inclusion complexes with a variety of guest molecules. Among the CDs evaluated, γ-CD emerged as the most effective carrier, capable of forming a hemi-inclusion complex with hemin via hydrogen bonding and hydrophobic interactions. This encapsulation technique markedly improved the dispersibility of hemin. Furthermore, the γ-CD/hemin inclusion complex exhibited reduced toxicity compared to FeSO_4_ in IEC-6 cells. In vitro digestion experiments, which mimic the gastrointestinal tract, have shown that the γ-CD/hemin complex can endure the harsh conditions of the stomach and maintain its stability. This suggests that only minimal amounts of hemin are absorbed in the gastric environment, whereas absorption is significantly higher in the intestinal tract. This points to the potential for the targeted intestinal delivery of hemin.

## Figures and Tables

**Table 1:** Encapsulation efficiency in different inclusion complexes.

Cyclodextrins	Encapsulation Efficiency (% ± SD, *n* = 3)
β-CD	70.91 ± 0.86 ^a^
Me-β-CD	70.24 ± 0.61 ^ab^
HP-β-CD	71.67 ± 1.23 ^a^
SBE-β-CD	68.17 ± 0.58 ^b^
γ-CD	72.11 ± 0.08 ^a^

Different lowercase letters (from “a” to “b”) behind the data represent the significance of the difference between the samples (*p* < 0.05).

**Table 2:** Solubility of hemin in different inclusion complexes.

Cyclodextrins	Hemin Solubility (μg/mL ± SD, *n* = 3)	Solubilization Ratio
β-CD	21.01 ± 1.82 ^e^	30.90
Me-β-CD	75.63 ± 4.49 ^d^	111.22
HP-β-CD	136.39 ± 0.80 ^c^	200.57
SBE-β-CD	151.84 ± 1.59 ^b^	223.29
γ-CD	163.61 ± 1.78 ^a^	240.60

Different lowercase letters (from “a” to “e”) behind the data represent the significance of the difference between the samples (*p* < 0.05).

## Data Availability

The original contributions presented in the study are included in the article, further inquiries can be directed to the corresponding author.

## References

[1] Conrad M.E., Cortell S., Williams H.L., Foy A.L. (1966). Polymerization and intraluminal factors in the absorption of hemoglobin-iron. J. Clin. Med..

[2] Vaghefi N., Nedjaoum F., Guillochon D., Bureau F., Arhan P., Bougle D. (2005). Iron absorption from concentrated hemoglobin hydrolysate by rat. J. Nutr. Biochem..

[3] Zhang Y., Zhao D., Xu J., Xu C., Dong C., Liu Q., Deng S., Zhao J., Zhang W., Chen X. (2013). Effects of Dietary Factors on the Pharmacokinetics of 58Fe-labeled Hemin after Oral Administration in Normal Rats and the Iron-deficient Rats. Biol. Trace. Elem. Res..

[4] Lin J., Chen X., Chen R., He L., Zheng L. (2000). Observation on the effect of hemin in improving human anaemia. J. Hyg. Res..

[5] Huang P., Yuan X., Hong Q., Lv F. (1995). Development and efficacy observation of hemin oral solution. J. Chin. Pharm. Sci..

[6] Deng X., Zhang N., Tang C. (2017). Soy protein isolate as a nanocarrier for enhanced water dispersibility, stability and bioaccessibility of -carotene. J. Sci. Food. Agric..

[7] Liu Q., Han C., Tian Y., Liu T. (2020). Fabrication of curcumin-loaded zein nanoparticles stabilized by sodium caseinate/sodium alginate: Curcumin solubility, thermal properties, rheology, and stability. Process Biochem..

[8] Yang J., Xiong L., Li M., Xiao J., Geng X., Wang B., Sun Q. (2019). Preparation and Characterization of Tadpole- and Sphere-Shaped Hemin Nanoparticles for Enhanced Solubility. Nanoscale Res. Lett..

[9] Churio O., Duran E., Guzman-Pino S.A., Valenzuela C. (2019). Use of Encapsulation Technology to Improve the Efficiency of an Iron Oral Supplement to Prevent Anemia in Suckling Pigs. Animals.

[10] Fan C., Yuan J., Guo J., Kang X. (2022). Soy protein isolate (SPI)-hemin complex nanoparticles as a novel water-soluble iron-fortifier: Fabrication, formation mechanism and in vitro bioavailability. Food Biosci..

[11] Span K., Verhoef J.J.F., Hunt H., van Nostrum C.F., Brinks V., Schellekens H., Hennink W.E. (2016). A novel oral iron-complex formulation: Encapsulation of hemin in polymeric micelles and its in vitro absorption. Eur. J. Pharm. Biopharm..

[12] Churio O., Pizarro F., Valenzuela C. (2018). Preparation and characterization of iron-alginate beads with some types of iron used in supplementation and fortification strategies. Food Hydrocoll..

[13] Valenzuela C., Hernandez V., Morales M.S., Neira-Carrillo A., Pizarro F. (2014). Preparation and characterization of heme iron-alginate beads. Lwt-Food Sci. Technol..

[14] Navas-Carretero S., Perez-Granados A.M., Sarria B., Vaquero M.P. (2009). Iron absorption from meat pate fortified with ferric pyrophosphate in iron-deficient women. Nutrition.

[15] Valdes F., Carrillo R., Campos F., Saenz L., Valenzuela C. (2023). Encapsulation of atomized erythrocytes in liposomes as source of heme iron for oral supplementation strategies. J. Food Process Eng..

[16] Churio O., Valenzuela C. (2018). Development and characterization of maltodextrin microparticles to encapsulate heme and non-heme iron. Lwt-Food Sci. Technol..

[17] Wang B., Cheng F., Gao S., Ge W., Zhang M. (2017). Double enzymatic hydrolysis preparation of heme from goose blood and microencapsulation to promote its stability and absorption. Food Chem..

[18] Cid-Samamed A., Rakmai J., Mejuto J.C., Simal-Gandara J., Astray G. (2022). Cyclodextrins inclusion complex: Preparation methods, analytical techniques and food industry applications. Food Chem..

[19] Mura P. (2015). Analytical techniques for characterization of cyclodextrin complexes in the solid state: A review. J. Pharm. Biomed. Anal..

[20] Saokham P., Muankaew C., Jansook P., Loftsson T. (2018). Solubility of Cyclodextrins and Drug/Cyclodextrin Complexes. Molecules.

[21] Li T., Guo R., Zong Q., Ling G. (2022). Application of molecular docking in elaborating molecular mechanisms and interactions of supramolecular cyclodextrin. Carbohydr. Polym..

[22] Pinho E., Grootveld M., Soares G., Henriques M. (2014). Cyclodextrins as encapsulation agents for plant bioactive compounds. Carbohydr. Polym..

[23] Yuan X., Hong Q., Lin G., Lin F. (2001). Development of hemin-βcyclodextrin inclusion complexes. Chin. Pharm..

[24] Bayat F., Homami S.S., Monzavi A., Olyai M.R.T.B. (2022). A combined molecular docking and molecular dynamics simulation approach to probing the host-guest interactions of Ataluren with natural and modified cyclodextrins. Mol. Simul..

[25] Gannimani R., Perumal A., Ramesh M., Pillay K., Soliman M.E., Govender P. (2015). Antipyrine-gamma cyclodextrin inclusion complex: Molecular modeling, preparation, characterization and cytotoxicity studies. J. Mol. Struct..

[26] Yousaf A.M., Qadeer A., Raza S.A., Chohan T.A., Shahzad Y., Din F.U., Khan I.U., Hussain T., Alvi M.N., Mahmood T. (2019). Influence of levodropropizine and hydroxypropyl-beta-cyclodextrin association on the physicochemical characteristics of levodropropizine loaded in hydroxypropyl-beta-cyclodextrin microcontainers: Formulation and in vitro characterization. Polim Med..

[27] Thomas C., Oates P.S. (2002). IEC-6 cells are an appropriate model of intestinal iron absorption in rats. J. Nutr..

[28] Ding B., Yu Y., Geng S., Liu B., Hao Y., Liang G. (2022). Computational Methods for the Interaction between Cyclodextrins and Natural Compounds: Technology, Benefits, Limitations, and Trends. J. Agric. Food. Chem..

[29] Gatiatulin A.K., Oselskaya V.Y., Klimovitskii A.E., Ziganshin M.A., Gorbachuk V.V. (2023). Influence of hydration and the size of the macrocycle of natice cyclodextrins on the solid phase inclusion of ritonavir. J. Struct. Chem..

[30] Yang B., Yang L., Lin J., Chen Y., Liu Y. (2009). Binding behaviors of scutellarin with α, β, γ-cyclodextrins and their derivatives. J. Incl. Phenom. Macrocycl. Chem..

[31] Yang X., Zhao Y., Chen Y., Liao X., Gao C., Xiao D., Qin Q., Yi D., Yang B. (2013). Host–guest inclusion system of mangiferin with β-cyclodextrin and its derivatives. Mater. Sci. Eng. C-Mater. Biol. Appl..

[32] Loh G.O.K., Tan Y.T.F., Peh K. (2016). Enhancement of norfloxacin solubility via inclusion complexation with β-cyclodextrin and its derivative hydroxypropyl-β-cyclodextrin. Asian J. Pharm. Sci..

[33] Singh S., Negi J.S., Bisht R., Negi V., Kasliwal N., Thakur V., Upadhyay A. (2014). Development and evaluation of orodispersible sustained release formulation of amisulpride–γ-cyclodextrin inclusion complex. J. Incl. Phenom. Macrocycl. Chem..

[34] Muankaew C., Jansook P., Stefansson E., Loftsson T. (2014). Effect of γ-cyclodextrin on solubilization and complexation of irbesartan: Influence of pH and excipients. Int. J. Pharm..

[35] Kapoor M.P., Moriwaki M., Ozeki M., Timm D. (2021). Structural elucidation of novel isoquercitrin-γ-cyclodextrin (IQC-γCD) molecular inclusion complexes of potential health benefits. Carbohydr. Polym. Technol. Appl..

[36] Wang Y., Cai M., Zeng H., Zhao H., Zhang M., Yang Z. (2022). Preparation, Characterization and Iron Absorption by Caco-2 Cells of the Casein Peptides-Iron Chelate. Int. J. Pept. Res. Ther..

